# Evaluation of Sporicidal Disinfectants for the Disinfection of Personal Protective Equipment During Biological Hazards

**DOI:** 10.1089/hs.2019.0128

**Published:** 2020-02-17

**Authors:** Stefanie Papp, Katharina Kimmerl, Jacob Gatz, Michael Laue, Roland Grunow, Oliver Kaspari

**Affiliations:** Dr. Stefanie Papp, Katharina Kimmerl, Jacob Gatz, Prof. Dr. Roland Grunow, and Dr. Oliver Kaspari are with the Centre for Biological Threats and Special Pathogens, Highly Pathogenic Microorganisms (ZBS 2); Dr. Michael Laue is with the Centre for Biological Threats and Special Pathogens, Advanced Light and Electron Microscopy (ZBS 4); all are with the Robert Koch Institute, Berlin, Germany.

**Keywords:** Disinfection, *Bacillus* spores, Biological hazards, Personal protective equipment

## Abstract

A fast, effective, and safe disinfection of personal protective equipment (PPE) is vitally important for emergency forces involved in biological hazards. This study aimed to investigate a broad range of disinfectants to improve the established disinfection procedure. We analyzed the efficacy of chlorine-, peracetic acid–, and oxygen-based disinfectants against *Bacillus* spores on PPE. Therefore, spores of different *Bacillus* species were exposed to disinfectants on PPE material by using a standardized procedure covering the dried spores with disinfectants and applying mechanical distribution. Efficacy of disinfectants was quantified by determining the reduction factor (log_10_ levels) and number of viable spores left afterward. The chlorine-based granulate Hypochlorit CA G (2% chlorine) sufficiently inactivated *Bacillus* spores of risk groups 1 and 2, even with temperatures ranging from −20 to 35°C. Wofasteril^®^ SC super (1.75% peracetic acid) achieved a reliable reduction of risk groups 1 and 2 and even fully virulent *Bacillus* spores by ≥5 log_10_ levels on PPE. With this, Hypochlorit-CA G and Wofasteril^®^ SC super proved to be promising alternatives to the previously proven and widely used peracetic acid compound Wofasteril^®^ (2% peracetic acid) for the disinfection of PPE when bacterial spores are known to be the contaminating agent. These results will help to improve the disinfection of PPE during biological hazards by providing new data on promising alternative compounds.

## Abstract 2

A fast, effective, and safe disinfection of personal protective equipment (PPE) is vitally important for emergency forces involved in biological hazards. This study aimed to investigate a broad range of disinfectants to improve the established disinfection procedure. The authors analyzed the efficacy of chlorine-, peracetic acid-, and oxygen-based disinfectants against Bacillus spores on PPE.

The management of incidents involving biological hazards, such as highly pathogenic bacteria, viruses, or toxins, bears a high risk of infection or contamination for emergency forces and civilians who are dealing with these biological agents.^1,2^ Besides naturally occurring hazards, like outbreaks of infectious diseases, possible intentional and accidental release of these biological agents could be of central importance for public health. These agents share a number of features with significant threats to human health: They have morbidity and lethality, they have high infectivity or toxicity, and they are difficult to treat, especially when recognized late. Moreover, they are suitable for mass production and delivery and may possess a high stability in the environment.

Therefore, in a case of an incident involving these agents, their dispersal must be limited, exposed people must be traced and treated, and contamination of the environment must be removed. Emergency forces must possess effective personal protective equipment (PPE) to avoid infection or contamination. Moreover, the fast and effective disinfection of PPE, pieces of evidence, and equipment is essential to prevent severe subsequent infections in biological risk situations.

Disinfectants used in scenarios of biological threats must fulfill specific requirements, like a high inactivation rate at a short exposure time, as well as a sufficient material and environmental compatibility.^3-6^ Additionally, a low-cost and easy-to-use product with minimal restrictions on storage and transportation would be of great interest. In recent years, various disinfection procedures have been established. In Germany, the Robert Koch Institute and the Federal Office of Civil Protection and Disaster Assistance have advised the use of a 2.0% peracetic acid solution combined with 0.2% surfactant with an exposure time of 5 minutes for the decontamination of PPE.^3,7^ Although peracetic acid has broad antimicrobial and also sporicidal activity, with low toxicity in comparison to other disinfectants like sodium hypochlorite or formaldehyde, the application for disinfecting PPE has several disadvantages.^8-10^ Besides the pungent odor, it can lead to irritation of the respiratory tract, eyes, and skin, even at low concentrations. With regard to disinfection of equipment, peracetic acid can corrode copper, brass, bronze, plain steel, and galvanized iron. Moreover, according to the Classes of Dangerous Goods (organic peroxides and corrosive substances), transportation is restricted, including prohibition of air transportation. Although peracetic acid decomposes to its original constituents, particularly if it is diluted, it is an environmentally hazardous substance and very toxic to aquatic organisms. Thus, protection of the environment, including waste management, must be considered.

Nevertheless, the application of effective disinfection procedures is essential in scenarios of biological threats with potentially high risk for public health. This is illustrated by a multitude of unfortunate events in the past, in which hazardous biological agents have been disseminated. Two of the most recent events in Germany occurred in 2012 and 2018, when a natural outbreak of anthrax in cattle in the district Stendal was detected and an attack with the toxin ricin was prevented in Cologne.^11^

Another alarming criminal incident of bioterrorism was the delivery of letters containing *Bacillus anthracis* spores in Washington, DC, in 2001.^2,12,13^ The potential use of spores as a bioweapon was also shown earlier when the religious cult Aum Shinrikyo aerosolized and released *B. anthracis* spores in Kameido, Japan, in 1993, and a further incident in Sverdlovsk in which spores were accidentally released from a Soviet military research facility in 1979.^2,14,15^ The highly pathogenic bacterium *B. anthracis* poses a particular danger in these situations. Very resistant spores can be produced, aerosolized, and disseminated, causing severe infections in humans and also in livestock and wildlife, which can lead to death.^2,16^ Spore formation is a special feature of Gram-positive *Bacillus* and *Clostridium* species, leading to dormant forms that survive environments of extreme temperature and low nutrients, as well as chemical treatment and UV radiation.^17,18^ Therefore, bacterial spores in particular pose high requirements on the efficiency of disinfectants.^3,5,6^

The present study aimed to improve the PPE disinfection procedure by finding an alternative product that possesses advantages but alleviates the disadvantages of 2% peracetic acid. We tested the efficacy against *Bacillus* spores on PPE, focusing on commercially available solid disinfectants (granulates), to address the problem of transportation restrictions.

Additionally, an aqueous disinfectant was also included in our study. The selected products were predominantly validated for the disinfection of surfaces or medical instruments by quantitative surface and suspension tests. For our tests, we used an already well-established standardized procedure to evaluate disinfection efficacy on PPE.^3,19^ Fluctuating ambient temperature was also taken into account, simulating disinfection of PPE at different temperatures.

## Materials and Methods

### Bacterial Strains

Spores of 6 *Bacillus* species (risk group [RG] 1 to 3) were used as bacterial contaminants on PPE material (Table 1). Spore preparation was performed on manganese sulfate agar according to DIN EN 14347:2005^20^ as described previously.^3,21^ Spores possessing an exosporium (all except *B. subtilis*) were suspended in 0.1% Triton X-100 (Carl Roth GmbH, Germany) in deionized water to prevent formation of spore aggregates. To obtain exact values of colony-forming units per ml (CFU ml^−1^), independent from spore aggregates, the CFU ml^−1^ calculation was performed by serial dilution using a mixture of 0.1% Triton X-100 in deionized water, followed by plating 100 μl of each dilution on tryptic soy agar (TSA) twice. Working concentrations of spores were adjusted to between 2 and 5x10^8^ CFU/ml^−1^.

**Table 1.** List of *Bacillus* Spores Used in This Study

Species	Strain	Origin	Biosafety Level	CFU ml^−1^	Resistance [log_10_ reduction] (0.05% paa)
*B. subtilis*	ATCC 6633	DSMZ Braunschweig, Germany	1	4.32x10^8^	6.57
*B. thuringiensis*	DSM 350	DSMZ Braunschweig, Germany	1	4.57x10^8^	1.86 ± 0.58
*B. cereus*	ATCC 12826	Pasteur Institute, France	2	2.84x10^8^	6.42 ± 0.01
*B. anthracis*	Sterne 34F2	W. Beyer, University of Hohenheim, Germany	2	5.09x10^8^	5.14^a^
	11/38	Institute for Consumer Health Protection and Veterinary Medicine (BgVV) Jena, Germany	3	1.66x10^8^	1.94
	22/39	Institute for Consumer Health Protection and Veterinary Medicine (BgVV) Jena, Germany	3	2.5x10^8^	2.52

DSMZ = German Collection of Microorganisms and Cell Cultures; paa = peracetic acid.

^a^ 0.01% paa.

### PPE Material and Carrier Preparation

PPE suit material was kindly provided by TESIMAX^®^, Altinger GmbH, Germany. Suit fabrics (carriers) in a size of 4 cm^2^ were prepared from the suits TESIMAX^®^ S3 PE-T and TESIMAX^®^ SYKAN 2. Prior to experiments, a circular test area was marked (2 cm^2^) on carriers that were then sterilized by UV irradiation with a dose of 4 J/cm^2^ on both sides.

### Disinfectants

Selection of disinfectants focused on commercially available solid (granulates) and on one aqueous disinfectant (Table 2). Preparations of working solutions were performed in sterile deionized water. Actual concentrations of chlorine in Hypochlorit-CA G and peracetic acid in Wofasteril^®^ SC super and Wofasteril^®^ were determined by iodometric titration prior to each experiment. To ensure proper wetting of hydrophobic PPE surfaces, different surfactants in deionized water were used as controls and mixed with disinfectants if not already included in the original formulation: This includes (1) Alcapur^®^ N (Kesla Hygiene AG, Germany) containing 45% sodium laureth sulphate, (2) Alcapur^®^ (Kesla Hygiene AG, Germany) containing 15% sodium hydroxide in water, and (3) sodium dodecyl sulphate (SDS, Carl Roth GmbH, Germany). Final concentrations of 0.5% Alcapur^®^ N (Wofasteril^®^), 0.5% Alcapur^®^ (Hypochlorit-CA G), 1.5% or 2.0% Alcapur^®^ (Wofasteril^®^ SC super), or 0.2% SDS (remaining disinfectants) were used.

**Table 2.** List of Disinfectants

Active Ingredient	Disinfectant	Manufacturer	Neutralization Medium
Chlorine			
	Hypochlorit-Ca G^a^	Meranus Gesellschaft für Schwimmbad- und Freizeitausrüstungen mbH	1% tryptic soy broth
	Chlorifix^a^	Bayrol Deutschland GmbH	neutralizer
	Halamid^®a^	Laboratorium Buchrucker Hygiene GmbH	neutralizer
Peracetic acid			
	Wofasteril^®^	Kesla Hygiene AG	neutralizer
	Wofasteril^®^ SC super	Kesla Hygiene AG	0.5% sodium sulfite in 1% tryptic soy broth
	Sekusept^®^ aktiv^a^	Ecolab Deutschland GmbH	neutralizer
	neodisher^®^ endo DIS active^a^	Chemische Fabrik Dr. Weigert GmbH & Co. KG	neutralizer
Oxygen			
	Dismozon^®^ plus^a^	Bode Chemie GmbH	neutralizer
	Perform^®a^	Schülke & Mayr GmbH	neutralizer
	Descogen^®^-I^a^	Antiseptica Dr. H.-J. Molitor GmbH	neutralizer
	Virkon^®^ S^a^	Antec International Limited (Sudbury, UK)	neutralizer

^a^ granulate; neutralizer = 3% tryptic soy broth, 9% (v/v) Tween 80, 0.9% (w/v) lecithin, and 3.0% (w/v) histidine.

### Neutralization of Disinfectants

To ensure exact exposure times, and by this exact determination of disinfection efficacy, 3 neutralization media were tested and validated depending on the disinfectant ingredient and its concentration (Table 2): (1) 1% tryptic soy broth (Oxoid, Germany), (2) 0.5% (w/v) sodium sulfite in 1% tryptic soy broth, or (3) 3% tryptic soy broth, 9% (v/v) Tween 80, 0.9% (w/v) lecithin, and 3.0% (w/v) histidine (Carl Roth GmbH, Germany).^4^ Validation of neutralization media was performed in 3 independent experiments prior to carrier assays according to DIN EN 14347:2005 using the dilution-neutralization method.^20^

### Test Method

Carrier assays for testing disinfection efficacy were performed using the “covering with mechanical action” technique described previously.^3,19^ In brief: 5 2-μl drops of a suspension containing between 2 and 5x10^8^ spores per ml were pipetted on the 2-cm^2^ circular test area (1 to 2.5x10^6^ spores cm^−2^) and dried for up to 45 minutes. To simulate disinfection of PPE on a small scale, 10 μl of disinfectant or control suspension (deionized water, with or without surfactant) was added. Disinfectants were then mechanically distributed onto the carriers with 2 inoculation loops for 30 seconds (“covering with mechanical action”), followed by an exposure for a total of 1, 3, 5, or 10 minutes.

The influence of temperature variation was determined by pre-heating or pre-cooling the disinfectants and incubation chambers to temperatures of interest for the time of disinfection. For experiments simulating harsh disinfection conditions at ambient temperature of −20°C, disinfectants were pre-cooled to 4°C, while the actual disinfection took place at −20°C.

Disinfection was stopped by having carriers transferred into 10 ml of neutralization medium, shaken for 10 minutes at 475 rpm, and incubated for 20 minutes at room temperature. Serial dilution was performed from the neutralization medium in 4.5 ml TSB, followed by double plating of 100 μl on TSA to precisely determine mean CFU 10 ml^−1^. For experiments under BSL-3 conditions, volumes of serial dilutions were decreased to 675 μl TSB. CFU ml^−1^ was determined after incubation for 24 and 48 hours at 37°C, respectively. To rule out delayed germination of spores, samples were again examined for growth after 7 days.

Sporicidal efficacy of a disinfectant is represented by the reduction factor (RF) and also by the number of remaining viable spores determined after 48 hours. The mean RF is calculated from 3 independent experiments (*n* = 3) with standard deviation. One experiment consisted of 2 identical experimental setups, and resulting CFU 10 ml^−1^ of both measurements were used to calculate the RF as follows:

Reduction factors were calculated by subtracting the CFU 10 ml^−1^ of the negative control (N_0_) from the CFU 10 ml^−1^ of samples mixed with disinfectant (N) (RF = log_10_N_0_ – log_10_N). A disinfectant is classified as efficient if viable spores are reduced by ≥5 log_10_ levels or more, meaning a reduction by a factor of at least 100,000 (gray horizontal bars in figures). Thus, based on the starting spore concentration, a survival of fewer than 10 to 25 spores cm^−2^ is required for a sufficient disinfection of PPE. In addition, CFU 10 ml^−1^ of negative control (N_0_) represents the maximally achievable reduction factor (log_10_N_0_) (dashed line in figures). If growth were detected in the liquid medium of the serial dilution but not on TSA plates, the reduction factor was calculated by using the maximum probable number of surviving bacteria in medium within the 95% confidence interval. This was done by integrating the probability distribution of each possible number of surviving bacteria for the used test parameters.

### Determination of Odor Intensity

Solutions of 2% chlorine (Hypochlorit-CA G / 0.5% Alcapur^®^), 1.75% and 2.75% peracetic acid (Wofasteril^®^ SC super/1.5% Alcapur^®^), and 2% peracetic acid (Wofasteril^®^/0.5% Alcapur^®^ N) were analyzed. Working solutions of 10 ml each were prepared in 100-ml glass bottles. As a control, solutions of 0.5% Alcapur^®^ N, 1.5% Alcapur^®^ in distilled water or pure distilled water were used. Fifteen subjects evaluated the odor intensity of each disinfectant, alternating with neutralizing coffee powder by chemical fanning. Odor intensity of disinfectants was determined referring to the regulation of olfactometry, ranging from 6 (extremely strong) to 0 (not perceptible).^22^

### Scanning Electron Microscopy (SEM)

As an internal experimental control, SEM was performed on the PPE carriers TESIMAX^®^ S3 PE-T and TESIMAX^®^ SYKAN 2 before and after treatment to exclude the influence of fabric damage on spore calculation. Small discs (5 mm in diameter) were punched out of the treated and untreated PSA samples using a tissue punch and fixed onto an SEM stub with conductive tape. A thin (5 nm) layer of gold/palladium was generated on the sample surface using a sputter coater (E5100, Polaron). Scanning microscopy was done with a tabletop microscope (TM3000, Hitachi High-Technologies) equipped with a semiconductor backscattered-electron detector at 5 and 15 kV acceleration voltage.

## Results

### Efficacy of Disinfectants

Pre-analyses of disinfectants revealed that only 2 of 10 tested disinfectants showed sufficient sporicidal efficacy against less resistant *B. subtilis* spores in our approach (data not shown). Eight disinfectants showed insufficient efficacy according to the specified requirements. In these cases, spore reduction was even below 2 log_10_ levels when testing manufacturer concentrations. Therefore, comparative analyses for the sporicidal efficacy against *Bacillus* spores on PPE were performed only with the chlorine-based granulate Hypochlorit-CA G, the peracetic acid–based disinfectant Wofasteril^®^ SC super, and the currently used Wofasteril^®^. Further pre-analyses showed that at least 1.5% chlorine (15,000 ppm) in a solution of Hypochlorit-CA G/0.5% Alcapur^®^ and 1.75% peracetic acid in a mixture of Wofasteril^®^ SC super/1.5% Alcapur^®^ are required to achieve a minimal sporicidal reduction by ≥5 log_10_ levels against resistant *B. thuringiensis* on PPE (data not shown).

**Figure 1.**
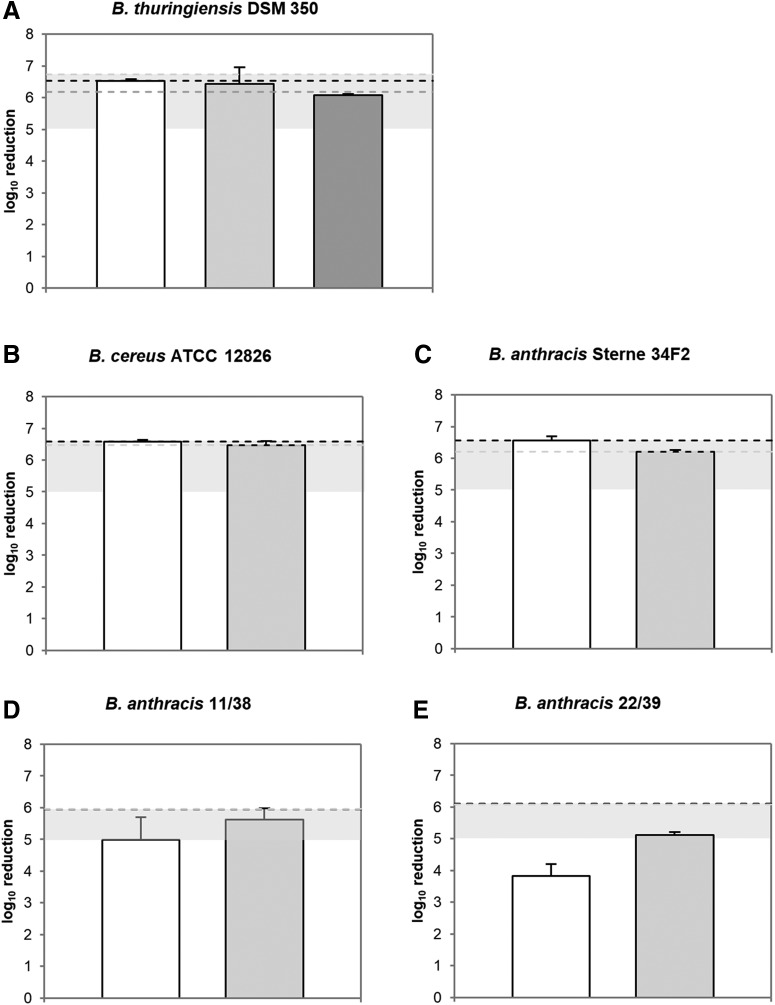
Efficacy of disinfectants against viable *Bacillus* spores. Hypochlorit-CA G/0.5 Alcapur^®^ (1.5% chlorine, white), Wofasteril^®^ SC super/1.5% Alcapur^®^ (1.75% peracetic acid, light grey), and Wofasteril^®^/0.5% Alcapur^®^ N (2% peracetic acid, dark grey) were tested using the “covering with mechanical action” technique on PPE material TESIMAX S3 PE-T with 5 minutes of contact time (*n* = 3). Graphs show log_10_ reduction of disinfectant against *B. thuringiensis* (A), *B. cereus* (B), or *B. anthracis* Sterne spores (C) and highly pathogenic *B. anthracis* 11/38 (D), or *B. anthracis* 22/39 spores (E) at room temperature with an organic burden of 0.3% BSA. Grey horizontal bars represent the range of successful disinfection. Dotted lines indicate maximal achievable reduction for each corresponding experimental approach.

These results were further used as starting concentrations for comparative testing of the 3 disinfectants. Experiments were performed using *Bacillus* spores of risk groups 1, 2, and 3 on PPE material TESIMAX^®^ S3 PE-T with an organic burden of 0.3% BSA at room temperature and 5 minutes of exposure time (Figure 1). Here, a chlorine concentration of 1.5% (Hypochlorit-CA G/Alcapur^®^) led to a full inactivation of *B. thuringiensis* spores with no remaining viable spores on PPE (Figure 1A; Table 3). In comparison to this, disinfection efficacy of Wofasteril^®^ SC super/Alcapur^®^ (1.75% peracetic acid) and Wofasteril^®^/Alcapur^®^ N (2% peracetic acid) was slightly impaired (6.44 ± 0.52 and 6.08 ± 0.04 log_10_ levels). Thus, a total number of ≥10 ± 14 and ≥1 ± 1 viable spores were able to germinate after the disinfection procedure.

**Table 3.** Disinfection Efficacy Against *Bacillus* Spores on PPE Material TESIMAX^®^ S3 PE-T Displayed by Spore Reduction [log_10_ level] and Total Number of Viable Spores

	Reduction [log_10_ level]	Total Number of Viable Spores
*B. thuringiensis*		
Hypochlorit-Ca G (1.5% chlorine)	≥6.54 ± 0.05	0
Wofasteril^®^ SC super (1.75% PES)	≥6.44 ± 0.52	≥10 ± 14
Wofasteril^®^ (2% PES)	≥6.08 ± 0.03	≥1 ± 1
*B. cereus*		
Hypochlorit-Ca G (1.5% chlorine)	≥6.58 ± 0.05	0
Wofasteril^®^ SC super (1.75% PES)	≥6.47 ± 0.13	0
*B. anthracis* Sterne		0
Hypochlorit-Ca G (1.5% chlorine)	≥6.56 ± 0.04	0
Wofasteril^®^ SC super (1.75% PES)	≥6.21 ± 0.13	0
*B. anthracis* 11/38		
Hypochlorit-Ca G (1.5% chlorine)	≥4.99 ± 0.70	≥10 ± 14
Wofasteril^®^ SC super (1.75% PES)	≥5.61 ± 0.36	≥10 ± 14
*B. anthracis* 22/39		
Hypochlorit-Ca G (1.5% chlorine)	≥3.83 ± 0.37	≥50 ± 14
Wofasteril^®^ SC super (1.75% PES)	≥5.12 ± 0.08	≥35 ± 31

However, analyses revealed a similar and sufficient sporicidal efficacy of these disinfectants against *B. thuringiensis*, indicated by an inactivation by at least 5 log_10_ levels. In addition, the efficacy of Hypochlorit-CA G/0.5% Alcapur^®^ and Wofasteril^®^ SC super/1.5% Alcapur^®^ was further tested against risk group 2 (Figure 1B and C) and highly pathogenic *Bacillus* spores (Figure 1D and E). Both disinfectants, with a concentration of 1.5% chlorine and 1.75% peracetic acid, achieved a full inactivation with no viable *B. cereus* and *B. anthracis* Sterne spores remaining (Figure 1B and C; Table 3). However, when testing against highly pathogenic *B. anthracis* 11/38 and 22/39 spores, efficacy of both disinfectants was impaired. The disinfection with 1.5% chlorine led to a reduction below 5 log_10_ levels (Figure 1D and E) with ≥10 ± 14 and ≥50 ± 14 remaining viable spores (Table 3). Although spores were exposed to a higher chlorine concentration of 2.5%, disinfection was only sufficient in the case of *B. anthracis* 11/38 spores with a reduction by ≥5.17 ± 0.73 log_10_ levels and ≥10 ± 14 remaining viable spores (Table 4). Disinfection of *B. anthracis* 22/39 spores resulted in an inactivation by only ≥4.52 ± 0.43 log_10_ levels. However, a reliable inactivation by ≥5.61 ± 0.36 and ≥5.12 ± 0.08 log_10_ levels was achieved against highly pathogenic *B. anthracis* 11/38 and 22/39 spores with 1.75% peracetic acid in Wofasteril^®^ SC super (Figure 1D and E), observing only ≥10 ± 14 and ≥35 ± 31 viable spores after disinfection.

**Table 4.** Disinfection Efficacy of Hypochlorit-CA G Against *Bacillus* Spores on PPE Material TESIMAX^®^ S3 PE-T Displayed by Spore Reduction [log_10_ level] and Total Number of Viable Spores

	Reduction [log_10_ level]	Total Number of Viable Spores
Hypochlorit-Ca G (2.5% chlorine)		
*B. anthracis* 11/38	≥5.17 ± 0.73	≥10 ± 14
*B. anthracis* 22/39	≥4.52 ± 0.43	≥50 ± 14

Given this observation, Hypochlorit-CA G/0.5% Alcapur^®^ (1.5% chlorine) and Wofasteril^®^ SC super/1.5% Alcapur^®^ (1.75% peracetic acid) efficiently inactivated *Bacillus* spores of risk groups 1 and 2 at room temperature within 5 minutes. Moreover, Wofasteril^®^ SC super, but not Hypochlorit-CA G, sufficiently reduced highly pathogenic *B. anthracis* spores.

### Efficacy Under Temperature Variation

**Figure 2.**
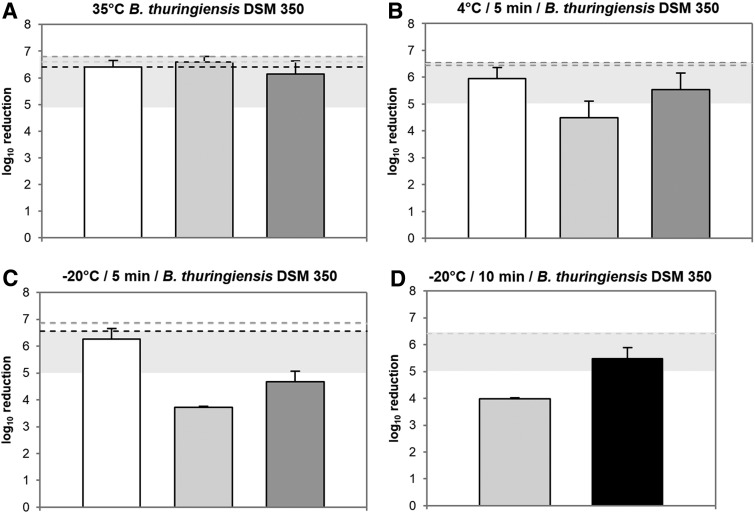
Efficacy of disinfectants against viable *B. thuringiensis* spores at different temperatures. Graphs show log_10_ reduction after treatment with Hypochlorit-CA G/0.5% Alcapur^®^ (1.5% chlorine, white), Wofasteril^®^ SC super/1.5% Alcapur^®^ (1.75%, light grey or 2.75% peracetic acid, black), or Wofasteril^®^/0.5% Alcapur^®^ N (2% peracetic acid, dark grey) using the “covering with mechanical action” technique on PPE material TESIMAX S3 PE-T (*n* = 3). Disinfection was performed at 35°C for 5 minutes (A), at 4°C for 5 minutes (B), and at −20°C for 5 minutes (C) or 10 minutes (D). Grey horizontal bars indicate the range of successful disinfection, while dotted lines show maximally achievable reduction for each corresponding experimental approach.

Efficacy of disinfectants was further tested on TESIMAX^®^ S3 PE-T at 35°C, 4°C, and −20°C and an exposure time of 5 to 10 minutes against *B. thuringiensis* spores (Figure 2; Table 5). At 35°C, a disinfection with 1.5% chlorine (Hypochlorit-CA G/0.5% Alcapur^®^) or 1.75% peracetic acid (Wofasteril^®^ SC super/1.5% Alcapur^®^) led to a full inactivation by ≥6.41 ± 0.25 and ≥6.60 ± 0.20 log_10_ levels with no remaining *B. thuringiensis* spores after 5 minutes of exposure time (Figure 2A; Table 5). In comparison, efficacy was impaired using 2% peracetic acid (Wofasteril^®^/0.5% Alcapur^®^ N). However, a sufficient inactivation by ≥6.14 ± 0.49 log_10_ levels with ≥35 ± 31 remaining viable spores was observed.

**Table 5.** Disinfection Efficacy Against *Bacillus* Spores on PPE Material TESIMAX^®^ S3 PE-T at Temperature from 35°C to −20°C Displayed by Spore Reduction [log_10_ level] and Total Number of Viable Spores

Disinfectant	Reduction [log_10_ level]	Total Number of Viable Spores
35°C/5 min		
Hypochlorit-Ca G (1.5% chlorine)	≥6.41 ± 0.25	0
Wofasteril^®^ SC super (1.75% PES)	≥6.60 ± 0.20	0
Wofasteril^®^ (2% PES)	≥6.14 ± 0.49	≥35 ± 31
4°C/5 min		
Hypochlorit-Ca G (1.5% chlorine)	≥5.94 ± 0.42	≥20 ± 14
Wofasteril^®^ SC super (1.75% PES)	≥4.48 ± 0.63	≥447 ± 459
Wofasteril^®^ SC super (2.75% PES)	≥6.22 ± 0.35	≥10 ± 14
Wofasteril^®^ (2% PES)	≥5.53 ± 0.62	≥109 ± 83
–20°C/5 min		
Hypochlorit-Ca G (1.5% chlorine)	≥6.27 ± 0.40	≥10 ± 14
Wofasteril^®^ SC super (1.75% PES)	≥3.72 ± 0.05	≥2333 ± 291
Wofasteril^®^ SC super (2.75% PES)	≥5.08 ± 0.05	≥109 ± 31
Wofasteril^®^ (2% PES)	≥4.67 ± 0.40	≥712 ± 301
–20°C/10 min		
Wofasteril^®^ SC super (1.75% PES)	≥3.99 ± 0.04	≥358 ± 110
Wofasteril^®^ SC super (2.75% PES)	≥5.48 ± 0.41	≥163 ± 121

A decline of temperature to 4°C or −20°C correlates with decreased efficacy of all 3 disinfectants against *B. thuringiensis* spores (Figure 2B and C; Table 5). However, 1.5% chlorine showed the most stable disinfection capacity. With an exposure time of 5 minutes, a spore reduction of 5.94 ± 0.42 log_10_ levels at 4°C and 6.27 ± 0.40 log_10_ levels even at −20°C was observed, leading to only ≥20 ± 14 and ≥10 ± 14 remaining viable spores.

In contrast, both peracetic acid–based disinfectants showed an incomplete spore inactivation. At 4°C, a solution of 1.75% peracetic acid (Wofasteril^®^ SC super/1.5% Alcapur^®^) failed to reduce *B. thuringiensis* spores by ≥5 log_10_ levels with ≥447 ± 459 remaining viable spores. Although 2% peracetic acid (Wofasteril^®^/0.5% Alcapur^®^ N) achieved a reduction of ≥5.53 ± 0.62 log_10_ levels on average (Figure 2B; Table 5), spores were not reliably inactivated above ≥5 log_10_ levels for all 3 experiments.

With regard to Wofasteril^®^ SC super/1.5% Alcapur^®^, an increased concentration of 2.75% peracetic acid at 4°C resulted in an enhanced disinfection capacity by ≥6.22 ± 0.35 log_10_ levels with ≥10 ± 14 remaining viable spores after 5 minutes (Table 5). At −20°C, however, 2.75% peracetic acid (Wofasteril^®^ SC super/1.5% Alcapur^®^) and 2% peracetic acid (Wofasteril^®^/0.5% Alcapur^®^ N) again failed to reliably reduce *B. thuringiensis* spores in 3 experiments (Figure 2C), leading to ≥109 ± 31 and ≥712 ± 301 remaining viable spores (Table 5). However, spore inactivation was improved for Wofasteril^®^ SC super/1.5% Alcapur^®^ to ≥5.48 ± 0.41 log_10_ levels with ≥163 ± 121 viable spores when contact time and peracetic acid content were increased to 10 minutes and 2.75% (Figure 2D; Table 5).

These data show that 1.5% chlorine in a solution of Hypochlorit-CA G/0.5% Alcapur^®^ achieved a reliable reduction of *B. thuringiensis* spores independent of temperature variation. In contrast, both peracetic acid compounds showed decreased efficacy when temperature was diminished. However, this was counteracted with increasing peracetic acid concentration and contact time of Wofasteril^®^ SC super.

### Efficacy on Different PPE Material

In addition to PPE material TESIMAX^®^ S3 PE-T (disposable), disinfectants were also tested on the PPE fabric TESIMAX^®^ SYKAN 2 (reusable). *B. thuringiensis* spores were spotted on samples of the 2 PPE materials and exposed to Hypochlorit-CA G and Wofasteril^®^ SC super for 5 minutes under various conditions (Table 6). Hypochlorit-CA G/0.5% Alcapur^®^ was analyzed with a concentration of 1.5% chlorine against *B. thuringiensis* spores with an organic load of 0.3% BSA and at 35°C or −20°C. Disinfection of *B. thuringiensis* spores on TESIMAX^®^ SYKAN 2 led to a sufficient reduction by more than 5 log_10_ levels for all tested conditions after 5 minutes. Compared with the inactivation on TESIMAX^®^ S3 PE-T material, disinfection tended to be less efficient.

**Table 6.** Efficacy of Disinfectants Against *B. thuringiensis* Spores Displayed by Spore Reduction [log_10_ level] and Total Number of Viable Spores on Different PPE Material

Disinfectant	Reduction [log_10_ level]		Total Number of Viable Spores	
	TESIMAX^®^ S3 PE-T	TESIMAX^®^ SYKAN 2	TESIMAX^®^ S3 PE-T	TESIMAX^®^ SYKAN 2
Hypochlorit-Ca G				
0.3% BSA^a^	≥6.54 ± 0.05	≥5.94 ± 0.37	≥0 ± 0	≥20 ± 14
35°C^a^	≥6.42 ± 0.25	≥5.74 ± 0.47	≥0 ± 0	≥27 ± 20
–20°C^a^	≥6.27 ± 0.40	≥5.66 ± 0.03	≥10 ± 14	≥30 ± 0
Wofasteril^®^ SC super				
0.3% BSA^b^	≥6.44 ± 0.52	≥5.66 ± 0.63	≥0 ± 0	≥50 ± 71
4°C^b^	≥4.48 ± 0.63	≥4.86 ± 0.47	≥447 ± 459	≥100 ± 20
4°C^c^	≥6.22 ± 0.35	≥6.58 ± 0.04	≥10 ± 14	≥0 ± 0

^a^ 1.5 % chlorine; ^b^ 1.75% peracetic acid; ^c^ 2.75% peracetic acid.

Peracetic acid at a concentration of 1.75% (Wofasteril^®^ SC super/2.0% Alcapur^®^) was tested at room temperature with an organic burden of 0.3% BSA on TESIMAX^®^ SYKAN 2. Disinfection resulted in a spore inactivation by more than 5 log_10_ levels within 5 minutes for both PPE materials. Additionally, 1.75% peracetic acid (Wofasteril^®^ SC super/2.0% Alcapur^®^) was analyzed at 4°C, resulting in no sufficient inactivation of *B. thuringiensis* spores on both fabrics within 5 minutes. An increase of peracetic acid to 2.75% restored disinfection capacity, leading to a reliable reduction of *B. thuringiensis* spores within 5 minutes.

Given this observation, comparable inactivation of *B. thuringiensis* spores with Hypochlorit-CA G/0.5% Alcapur^®^ and Wofasteril^®^ SC super/1.5% Alcapur^®^ was observed on both PPE fabrics TESIMAX^®^ SYKAN 2 and TESIMAX^®^ S3 PE-T.

### Stability of Active Ingredients and Odor

**Figure 3.**
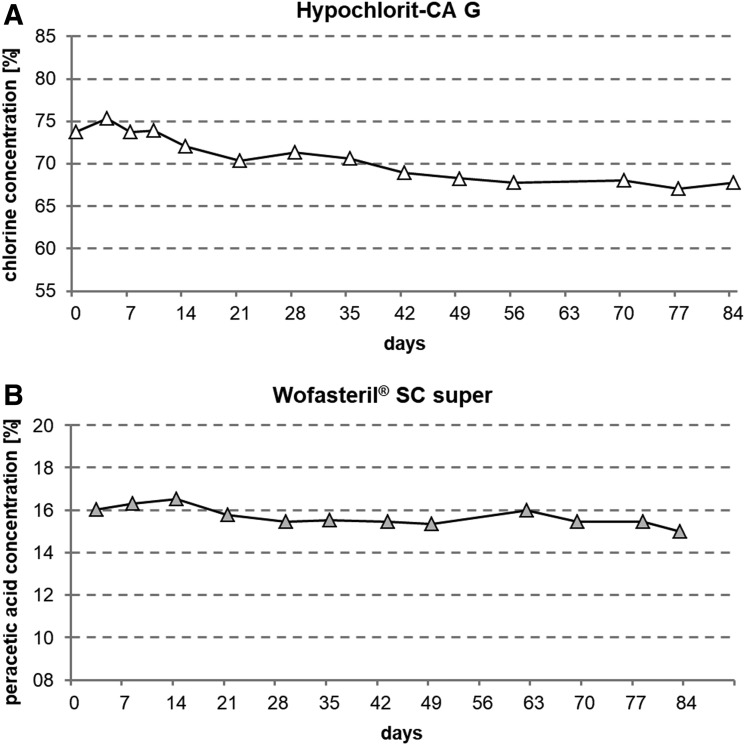
The stability of active ingredients in Hypochlorit-CA G, Wofasteril^®^ SC super. Concentration of chlorine in Hypochlorit-CA G (A) and peracetic acid in Wofasteril^®^ SC super (B) was determined by iodometric titration over 12 weeks. Each symbol represents 3 measurements at 1 point in time.

To analyze the stability of the active ingredients in Hypochlorit-CA G and Wofasteril^®^ SC super, chlorine and peracetic acid concentrations were determined over several weeks by iodometric titration (Figure 3). At the time of opening, Hypochlorit-CA G showed a free chlorine concentration of 74% (≙ 736,944 ppm) in accordance with the manufacturer's specifications of ≥70%, and this concentration was monitored over 84 days (Figure 3A). Chlorine concentration dropped continuously and fell below 70% after 42 days and reached 68% (≙ 677,894 ppm) after 84 days. This corresponds to a total loss of free chlorine by 8% in this period of time. Peracetic acid concentration in Wofasteril^®^ SC super was determined in the same manner (Figure 3B). According to the manufacturer's specifications, this disinfectant contains 11.0% to 15.0% peracetic acid. A concentration of 16% was determined 3 days after opening. The concentration of peracetic acid dropped to 15% after 84 days. A loss of 1% of peracetic acid, and thus a total loss of 6.5%, was determined within 84 days and so did not drop below manufacturer's specifications within 3 months after opening.

The “odor intensity” of the disinfectant solutions Hypochlorit-CA G/0.5% Alcapur^®^ (2% chlorine), Wofasteril^®^ SC super/1.5% Alcapur^®^ (1.75% and 2.75% peracetic acid) and Wofasteril^®^/0.5% Alcapur^®^ N (2% peracetic acid) was evaluated (data not shown). A solution of 2% chlorine tended to have the lowest odor intensity, followed by 1.75% peracetic acid in Wofasteril^®^ SC super and 2% peracetic acid in Wofasteril^®^. The strongest odor was perceived at 2.75% peracetic acid in Wofasteril^®^ SC super.

### Surface Structure of Treated PPE Material

Visual inspection of PPE material surfaces was performed by scanning electron microscopy (SEM) before and after treatment with UV radiation (4 J/cm^2^), as well as after treatment with 2.5% chlorine (Hypochlorit-CA G/0.5% Alcapur^®^), 2.75% peracetic acid (Wofasteril^®^ SC super/1.5% Alcapur^®^) and 2% peracetic acid (Wofasteril^®^/0.5% Alcapur^®^ N) to observe possible fabric damage that might influence spore inactivation (Figures S1 and S2; see supplemental material at https://www.liebertpub.com/doi/suppl/10.1089/hs.2019.0128).

TESIMAX^®^ S3 PE-T and TESIMAX^®^ SYKAN 2 surfaces exhibited no difference after UV irradiation in comparison to untreated PPE material. Furthermore, none of the applied disinfectants led to a structural alteration of the material surface. However, on TESIMAX^®^ S3 PE-T surface, patches of thin-layered adhesions were observed, which frequently showed cracks, possibly introduced by dried unconsumed hypochlorite (Ca(CCl)_2_).

## Discussion

The disinfection of pathogenic agents on personal protective equipment is of vital importance in dealing with biological hazards. This study intended to analyze the efficacy of disinfectants for the disinfection of PPE suits to find an alternative product for the currently used peracetic acid compound (Wofasteril^®^ (2% peracetic acid)/0.2% SDS). Altogether, in this study, 10 commercially available disinfectants were analyzed. We focused on a chlorine-based granulate Hypochlorit-CA G and the peracetic acid–based liquid compound Wofasteril^®^ SC super.

The efficacy of a disinfectant depends primarily on its active ingredient, its concentration, application time, and the volume used. In addition, the appropriate disinfection procedure for the intended use, in combination with the target organism, affects the outcome. In this study, chemicals that were not able to reduce *B. subtilis* spores sufficiently (by at least 5 log_10_ levels) were considered as not effective for our approach. Here, 8 of the 10 tested disinfectants showed insufficient inactivation of risk group 1 *B. subtilis* spores according to the specified requirements.^3^

However, this does not imply that these compounds are not effective against spores per se or moreover for their intended use. Selected granulates were previously tested, tailored to their purposes. This includes several protocols regarding quantitative surface and suspension tests provided by the Robert Koch Institute,^23^ the German Association for Applied Hygiene,^24^ and the German Institute for Standardization.^25^ They predominantly focus on instrument and surface disinfection and impose high requirements concerning the field of human and veterinary hygiene and health or water care.

The procedure used in our experiments, on the other hand, poses very high demands for the disinfection of PPE during biological hazards, concerning the amount of liquid, disinfection time, materials, microbial contaminants, and the procedure itself.^3,19^ Although procedures specified by manufacturers are partly intended to be used for testing disinfection by covering surfaces with an excess of liquid or foam, their parameters deviate for all selected disinfectants from the requirements of the “covering with mechanical action” procedure we used in our study. For this reason, a comparison with other studies is quite difficult. In consultation with the German Federal Office of Civil Protection and Disaster Assistance, an application time of 10 minutes was selected as the maximum endurable time for a person in PPE to stand still in a decontamination tent, waiting for the disinfectant to take effect. Furthermore, a reduction by at least 5 log_10_ levels had to be achieved.

In contrast, exposure times recommended by the manufacturers can range from 15 minutes to 4 hours to achieve antimicrobial efficacy.^23,24^ Moreover, the procedure used in the present study permits only the use of highly resilient *Bacillus* spores, whereas less stable vegetative bacteria and *Clostridium difficile* spores are used in test methods specified by the manufacturers. Only 1 of the granulates, Sekusept^®^ active, was previously shown to be effective against *B. subtilis* spores (≥3 log levels) according to DIN EN 13704^25^ and the limitations of a suspension test.^26^ However, some of these chemicals, which proved to be insufficient in the model used here, could be suitable for suspension disinfection during biological hazard events as is normally performed with pieces of evidence or equipment that are not part of PPE. This approach is of great interest and will be addressed in future studies.

On the other hand, 2 of 10 disinfectants, Hypochlorit-CA G and Wofasteril^®^ SC super, showed a promising spore inactivation on PPE in our study. Concerning the efficacy of Hypochlorit-CA G, the presented results are hard to discuss due to the lack of comparable studies. However, previous observations of chlorine-based disinfectants have shown sporicidal activity against *B. subtilis* and *B. anthracis* spores.^3,27,28^ Due to their broad antimicrobial activity and sporicidal efficacy, a sodium hypochlorite solution with ≥5,000 ppm free chlorine is recommended to be used in biological hazards by the World Health Organization (WHO),^29^ the US Centers for Disease Control and Prevention (CDC),^10^ and the US National Response Team.^30^ Although promising results were found with regard to *B. thuringiensis*, *B. cereus*, and *B. anthracis* Sterne, fully virulent spores of *B. anthracis* could not be sufficiently reduced by at least 5 log_10_ levels in our experiments, even when the chlorine concentration was increased to 2.5% (25,000 ppm). Different causes have to be considered, such as resistance and spore clumping. Environmental isolates of *B. anthracis* spores often differ from laboratory strains and are known to sporulate faster and to be more resilient than laboratory strains.^31^ Interestingly, *B. anthracis* 11/38 and 22/39 showed resistance against peracetic acid comparable to *B. thuringiensis* spores in prior experiments, while *B. anthracis* Sterne was much more susceptible (data not shown).

However, it has to be stated that the resistance of *B. anthracis* spores and spores in general vary widely in the literature, and there are also sources that did not observe a significant difference in resilience against peracetic acid between wild isolates and laboratory strains.^32^ Another special feature of *Bacillus* spores is the formation of spore aggregates due to their hydrophobic spore surface.^17,33^ This might influence disinfection efficacy due to shielding against chemicals, as it was found for heat treatment^34^ and would be an interesting avenue for future analyses. Although the aggregation of spores was counteracted by addition of 0.1% Triton X-100, remaining aggregates and re-aggregation of spores during the procedure cannot be excluded. Interestingly, the sporicidal effect of the tested granulate was unaffected by protein contamination even though chlorine solutions are known to be rapidly neutralized by organic matter.^35^

The use of solid disinfectants (granulates), like Hypochlorite-CA G, for the disinfection of PPE in the field would offer several advantages with regard to storage and transportation, since they would not have to be constantly cooled and are easy to prepare. However, it has to be kept in mind that the risk of chlorine release increases above 35°C according to the manufacturer's specifications. On the other hand, the odor of the working solution of Hypochlorit-CA G was perceived as less unpleasant than a peracetic acid solution of Wofasteril^®^ and Wofasteril^®^ SC super by the majority of test participants in this study. Even if no solid disinfectant proved to be unrestrictedly recommendable, the peracetic compound Wofasteril^®^ SC super showed high potential to be used as an alternative to the disinfectant Wofasteril^®^ currently applied by fire brigades when dealing with biological hazards (2% peracetic acid mixed with 0.2% SDS).^7^ The antimicrobial and sporicidal properties of peracetic acid are well and long known.^4,19,36-39^ In the present study, Wofasteril^®^ SC super/1.5% Alcapur^®^ was able to achieve a *B. anthracis* spore reduction by at least 5 log_10_ levels with a peracetic concentration of 1.75% within 5 minutes. This is in line with previous observations, where 2% peracetic acid in Wofasteril^®^/0.5% Alcapur^®^ N inactivated *B. anthracis* spores within 3 minutes.^3,21^ Temperatures of 4°C or below reduced its sporicidal efficacy below 5 log_10_ levels, a level that was less for Wofasteril^®^. In contrast, no impact of temperature decrease was observed previously with *B. subtilis* spores,^3^ underlining the impact of resistance on disinfectant efficacy. However, spore inactivation above 5 log_10_ levels was achieved with 2.75% peracetic acid (Wofasteril^®^ SC super) after 10 minutes at −20°C, whereas Wofasteril^®^ (2% peracetic acid) did not reach sufficient inactivation.

Another important observation was that no material alterations of the test carriers by UV radiation, peracetic acid, and chlorine could be observed by SEM (Figures S1 and S2). This was very important to determine, since surface damage by UV (sterilization method) of the test carriers would presumably make the material more porous and thus falsify the experiments. Naturally, damage of the fabric by the disinfectant also had to be excluded.

Peracetic acid content was very stable over 84 days, even when the bottle had already been opened. Furthermore, the addition of 1.5% Alcapur proved to be very efficient, which would negate the need for SDS, which represents a hazardous substance by itself. Thus, it is subject to transport regulations and precipitates at low temperatures. Moreover, Wofasteril^®^ SC super is toxicologically safe, since it disintegrates to hydrogen peroxide, acetic acid, and oxygen. Finally, the odor of Wofasteril^®^ SC super containing 1.75% peracetic acid was perceived as less unpleasant than 2% peracetic acid in the normally used Wofasteril^®^ by the majority of test participants. This product showed high inactivation capacity for *Bacillus* spores, but inactivation of viruses and toxins was not tested in this study. Further studies with other pathogens would be of great interest regarding the management of biological risk situations, especially when hazardous agents are unknown. However, 2% peracetic acid (Wofasteril^®^/0.5% Alcapur^®^ N) showed efficacy against viruses and toxins in a previous study,^3^ which should encourage future analyses of Wofasteril^®^ SC super to validate this disinfectant for a general deployment in those situations.

Taken together, our results indicate that Wofasteril^®^ SC super might be a suitable alternative disinfectant for the presently used Wofasteril^®^ if the exposition is likely restricted to bacterial spores. The solid disinfectant Hypochlorit-CA G could prove to be a suitable alternative, especially at low temperatures. However, it cannot be unreservedly recommended for this purpose due to its less reliable inactivation of bacterial spores.

## Supplementary Material

Supplemental data
