# Supplementary material for: Evaluation of Sporicidal Disinfectants for the Disinfection of Personal Protective Equipment During Biological Hazards
Source: Health Secur. 2020 Feb 17;18(1):36–48. doi: 10.1089/hs.2019.0128 (PMC7047094; doi:10.1089/hs.2019.0128)
Supplement: Supplemental data [file Supp_FigS1-S2.pdf]

Supplemental Material

Figure S1 and S2. Visualization of PPE material surfaces by scanning electron microscopy. TESIMAX® S3 PE-T (Figure S1) and TESIMAX® SYKAN 2 (Figure S2) were analyzed before and after UV radiation (4 J/cm^2), or treatment with Hypochlorit-CA G/0.5% Alcapur® (2.5% chlorine), Wofasteril® SC super/1.5% Alcapur® (2.75% peracetic acid), or Wofasteril®/0.5% Alcapur® N (2% peracetic acid) for 10 minutes. TESIMAX® S3 PE-T shows a smooth surface, which is, in the case of the chemically treated samples, regionally covered by a thin layer of material not present in untreated controls. It is of note that this additional material shows cracks but the PPE material surface does not. TESIMAX® SYKAN 2 reveals a more granular surface with tiny holes (smaller than 5 µm maximum diameter) in the upper layers of the surface, which is not significantly impaired by UV or treatment with the disinfectants.

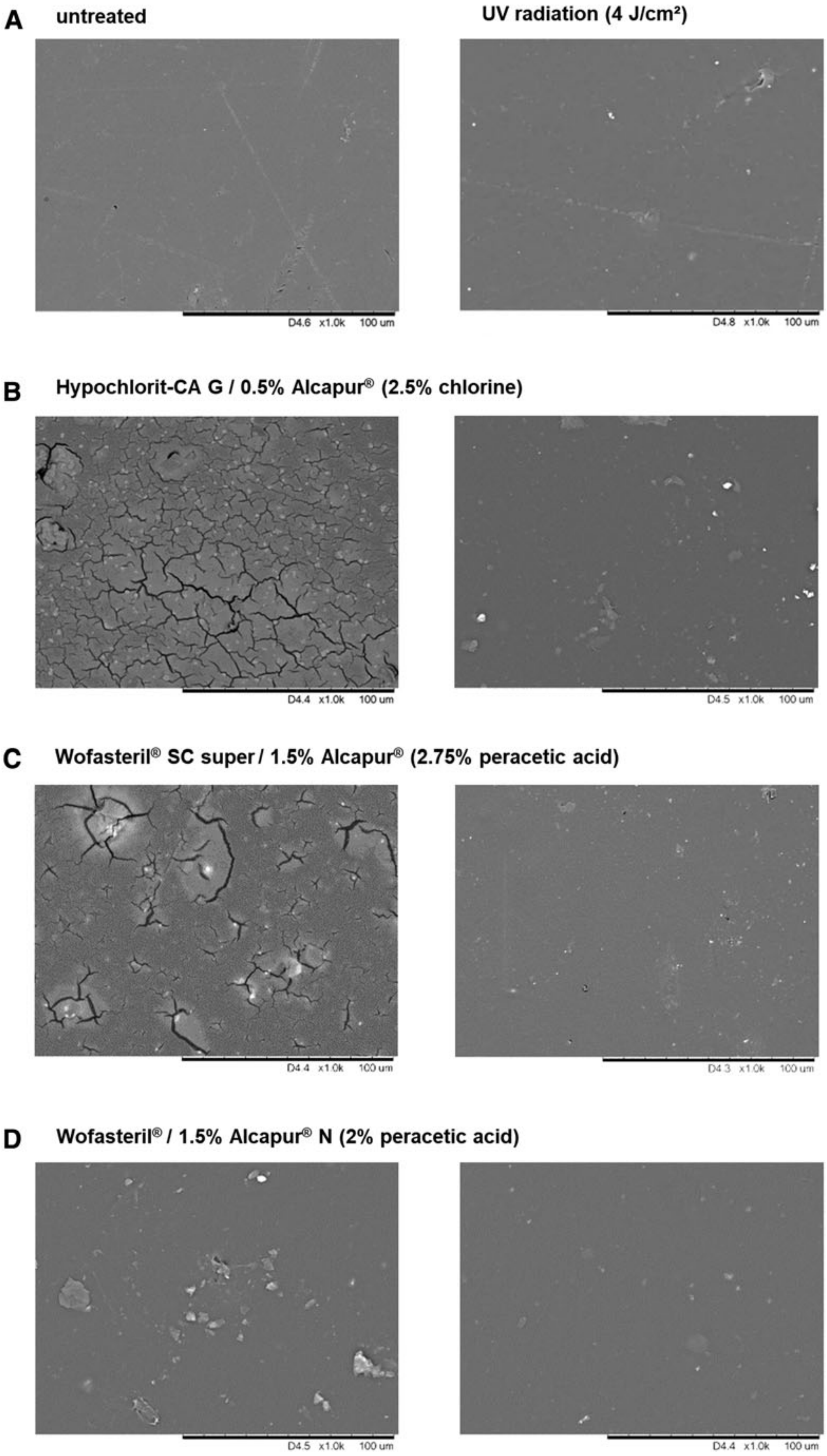

Supplementary Figure S1.

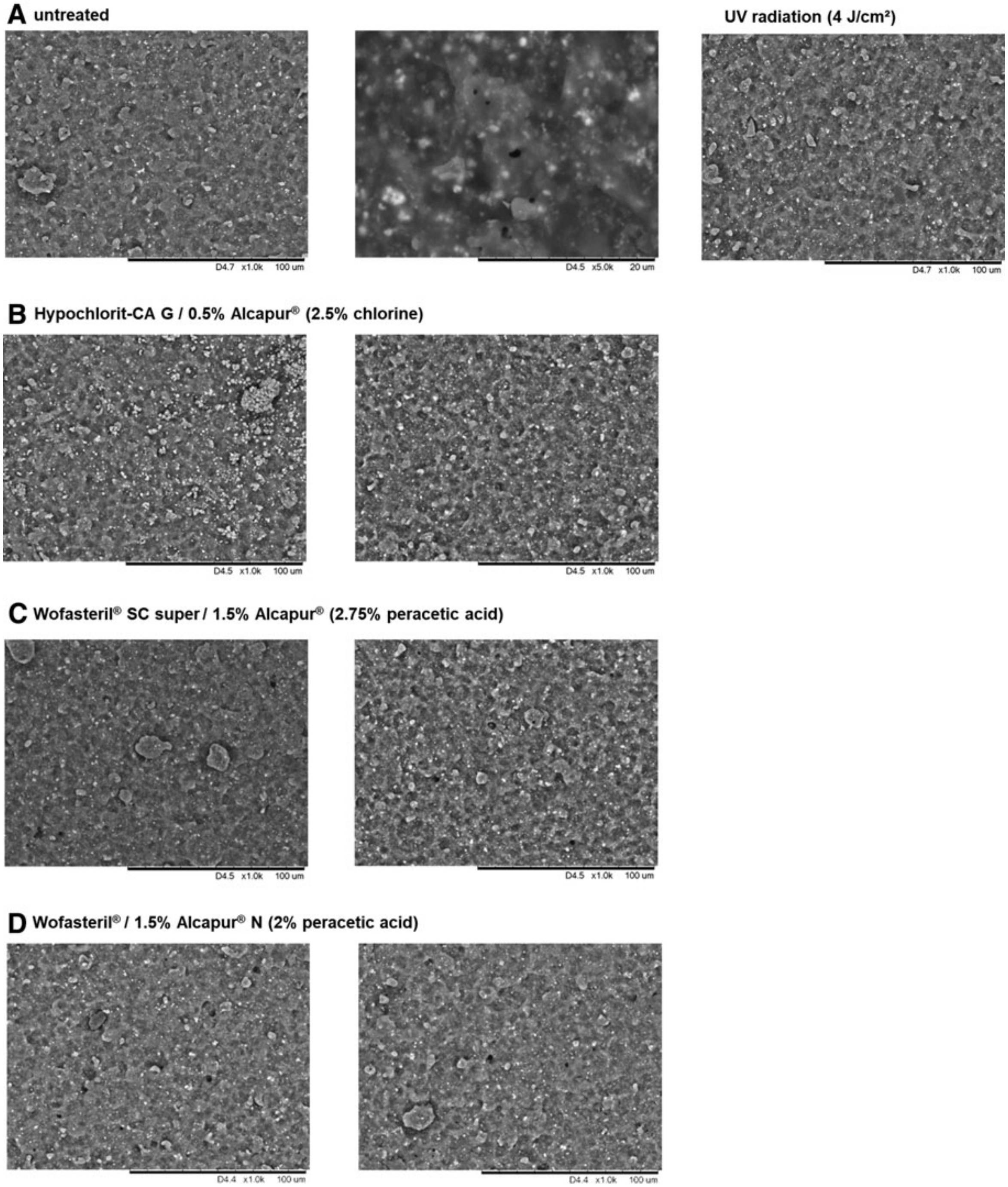

Supplementary Figure S2.
